# Administration of Parenteral Vitamin C in Patients With Severe Infection: Protocol for a Systematic Review and Meta-analysis

**DOI:** 10.2196/33989

**Published:** 2022-01-06

**Authors:** Arnav Agarwal, John Basmaji, Shannon M Fernando, Fang Zhou Ge, Yingqi Xiao, Haseeb Faisal, Kimia Honarmand, Mathieu Hylands, Vincent I Lau, Kimberley Lewis, Rachel Couban, François Lamontagne, Neill KJ Adhikari

**Affiliations:** 1 Division of General Internal Medicine Department of Medicine McMaster University Hamilton, ON Canada; 2 Division of Critical Care, Department of Medicine Schulich School of Medicine and Dentistry Western University London, ON Canada; 3 Division of Critical Care Department of Medicine University of Ottawa Ottawa, ON Canada; 4 Department of Emergency Medicine University of Ottawa Ottawa, ON Canada; 5 Michael G DeGroote School of Medicine McMaster University Hamilton, ON Canada; 6 Department of Health Research Methods Evidence and Impact McMaster University Hamilton, ON Canada; 7 Department of Nursing, West China School of Nursing West China Hospital Sichuan University Chengdu China; 8 Faculty of Medicine McMaster University Hamilton, ON Canada; 9 Interdepartmental Division of Critical Care Medicine University of Toronto Toronto, ON Canada; 10 Department of Critical Care Medicine Faculty of Medicine and Dentistry University of Alberta Edmonton, AB Canada; 11 Department of Medicine McMaster University Hamilton, ON Canada; 12 Department of Anesthesia McMaster University Hamilton, ON Canada; 13 Department of Medicine Université de Sherbrooke Sherbrooke, QC Canada; 14 Centre de Recherche du Centre Hospitalier Universitaire de Sherbrooke Sherbrooke, QC Canada; 15 Department of Critical Care Medicine Sunnybrook Health Sciences Centre Toronto, ON Canada

**Keywords:** vitamin C, ascorbic acid, severe infection, sepsis, COVID-19, SARS-CoV-2, infection, parenteral, vitamin, infectious disease, protocol, review, meta-analysis, treatment, inflammation, oxidation, effectiveness, safety, critical care

## Abstract

**Background:**

Severe infections are characterized by inflammation and oxidative damage. Ascorbic acid (vitamin C) administration may attenuate oxidative damage and, in turn, reduce vascular endothelial injury in pulmonary and systemic vasculature.

**Objective:**

We aim to describe a protocol for a living systematic review that will evaluate the effectiveness and safety of parenteral vitamin C administration in adults with severe infections, including those with COVID-19.

**Methods:**

We searched Ovid MEDLINE, Embase, CINAHL, the Centers for Disease Control and Prevention COVID-19 database, Cochrane Central Register of Controlled Trials, and ClinicalTrials.gov from inception to March 30, 2021, for randomized controlled trials evaluating parenteral vitamin C versus no parenteral vitamin C in hospitalized adults with severe infection. Eligible studies will include at least 1 arm involving any dose of parenteral vitamin C alone or in combination with other cointerventions and at least 1 arm not involving parenteral vitamin C. The primary outcomes of interest will include in-hospital, 30-day, and 90-day mortality. Title and abstract screening, full-text screening, data extraction, and risk of bias evaluation via a modified Risk of Bias 2.0 tool will be conducted independently and in pairs. We will perform random effects modeling for meta-analyses, in which study weights will be generated by using the inverse variance method. We will assess certainty in effect estimates by using the Grading of Recommendations Assessment, Development and Evaluation methodology. Meta-analyses will be updated iteratively as new trial evidence becomes available.

**Results:**

Among the 1386 citations identified as of March 30, 2021, a total of 17 eligible randomized controlled trials have been identified as of September 2021. We are in the process of updating the search strategy and associated data analyses.

**Conclusions:**

The results will be of importance to critical care physicians and hospitalists who manage severe infection and COVID-19 in daily practice, and they may directly inform international clinical guidance. Although our systematic review will incorporate the most recent trial evidence, ongoing trials may change our confidence in the estimates of effects, thereby necessitating iterative updates in the form of a living review.

**Trial Registration:**

PROSPERO CRD42020209187; https://www.crd.york.ac.uk/prospero/display_record.php?RecordID=209187

**International Registered Report Identifier (IRRID):**

RR1-10.2196/33989

## Introduction

Severe infections may be associated with intense inflammation and oxidative stress, which result in endothelial damage. Suspected or confirmed infection, together with a dysregulated host immune response causing acute organ dysfunction, as defined by the sepsis-related organ failure assessment (SOFA) score [1], defines sepsis [2]. Recent estimates from the Global Burden of Disease Study reported an estimated 48.9 million (95% uncertainty interval: 38.9 million to 62.9 million) incident cases of sepsis worldwide in 2017, along with 11.0 million (95% uncertainty interval: 10.1 million to 12 million) related deaths (19.7% of all-cause mortality worldwide) [3]. Interventions targeting the dysregulated host immune response and endothelial injury associated with severe infections and sepsis may improve patient-important outcomes [3-5]. For example, corticosteroids have been associated with improved survival in both sepsis [6-8] and severe COVID-19 [9-11].

Ascorbic acid deficiency, or vitamin C deficiency, has been reported in patients with severe infection [12,13], with preclinical data lending credence to the use of vitamin C in sepsis. Supplementation with vitamin C has been postulated to reduce vascular endothelial injury in pulmonary and systemic vasculature and reduce oxidative damage, and vitamin C has been postulated to act synergistically with glucocorticoids to suppress inflammation [14]. Due to the potential pulmonary effects associated with parenteral vitamin C and the preliminary evidence suggesting that vitamin C deficiency can occur in patients with SARS-CoV-2 infection [15], the World Health Organization (WHO) has prioritized parenteral vitamin C administration as a candidate treatment for COVID-19 as well [16].

Randomized controlled trials (RCTs) evaluating the effectiveness of vitamin C supplementation, either alone or in combination with other therapies such as corticosteroid and thiamine therapy, have yielded varied results. A systematic review in 2019 included RCTs of oral and parenteral vitamin C administration as monotherapy or in combination with other interventions in critically ill patients. The review found 9 RCTs that showed no significant overall survival benefit (risk ratio [RR] 0.72, 95% confidence interval [CI] 0.43-1.20) and no significant differences in the risk of new infections, length of intensive care or hospital stay, or duration of invasive mechanical ventilation. The subgroup analysis revealed a possible survival benefit with intravenous high-dose vitamin C monotherapy (RR 0.21, 95% CI 0.04-1.05), but the certainty of the evidence was limited by a small number of trials, patients, and events [17].

Several recent RCTs have evaluated parenteral vitamin C in the context of various populations of patients with sepsis [18-20]. Our systematic review and meta-analysis will aim to assess the efficacy and safety of parenteral vitamin C administration as monotherapy or in combination with other therapies in adult patients with severe infection.

## Methods

This protocol adheres to the Preferred Reporting Items for Systematic Reviews and Meta-Analysis Protocols (PRISMA-P) standards (Multimedia Appendix 1).

### Search Strategy

With the aid of a medical librarian, we searched the following electronic databases from inception to March 30, 2021: Ovid MEDLINE Daily and MEDLINE (from 1946), Embase (from 1974), CINAHL (from 1984), Cochrane Central Register of Controlled Trials, and ClinicalTrials.gov (Multimedia Appendix 2). A combination of keywords and medical subject heading terms were used, as follows: *ascorbic acid*, *vitamin C*, *sepsis*, *septic shock*, *infection*, *multiple organ failure*, *critical illness*, *intensive care unit*, *severe illness*, *respiratory distress syndrome*, *parenteral nutrition*, and *intravenous administration*. No language restrictions were applied.

We also searched the US Centers for Disease Control and Prevention COVID-19 database from inception to September 10, 2021. Specifically, we imported all Centers for Disease Control and Prevention COVID-19–related articles into EndNote (Clarivate Analytics) and conducted keyword searches with the terms *vitamin* or ascorb*. We will screen these studies to identify eligible studies by using a predeveloped search filter. We will also include additional eligible studies, which will be identified by the review authors and clinical experts. We did not search grey literature, but we will include the conference abstracts and unpublished data that were retrieved in our searches (eg, unpublished data retrieved from ClinicalTrials.gov).

To maintain currency, we will adopt a living evidence framework and will update the search every 2 months.

### Eligibility Criteria

We will include studies meeting the eligibility criteria described below.

#### Design and Population

We will include parallel-arm RCTs of adults aged ≥18 years with severe infection. Severe infection will be defined as the presence of suspected or microbiologically confirmed infection for which hospital ward or intensive care unit admission would be considered appropriate (ie, if feasible with available resources). We intentionally chose a liberal characterization of severe infection to include associated end-organ dysfunction (ie, sepsis [2]) and other studies in which organ dysfunction is not reported but patients are acutely ill and may still benefit from early treatment. We also chose this approach to account for the lack of intensive care units in many resource-limited settings [21] and variability in clinical practices across settings. Eligible studies may also enroll patients with severe infection involving at least 2 quick SOFA (qSOFA) criteria [22] or those meeting the WHO definition of sepsis (systolic blood pressure: <90 mm Hg) and 1 or more of the following: a pulse of >100 beats per minute, a respiratory rate of >24 breaths per minute, or abnormal temperature (<36 °C or >38 °C) [23]. We chose qSOFA criteria given feasibility of application across settings independent of laboratory criteria, despite imperfect discrimination of mortality in infected patients [24]. We will also consider the following studies to be eligible: (1) those in which at least 80% of patients meet our inclusion criteria for the population of interest and (2) those that include patients with severe organ failure, such as acute respiratory distress syndrome (ARDS), as long as outcomes are reported for the population with severe infection as the risk factor for ARDS.

#### Intervention

Trials with at least 1 arm involving the administration of parenteral vitamin C alone or in combination with other micronutrients and therapies will be included. Eligible studies will include those that administered doses of parenteral vitamin C above the very small doses that are typically seen in parenteral nutrition settings (100 mg/day), including high doses (>12 g/day), moderate doses (6-12 g/day), and low doses (<6 g/day). These dose cut points are arbitrary, but the use of 6 g to define the lower bound of high doses is consistent with a previous scoping review of adverse effects [25]. For studies using a weight-based regimen of vitamin C, we will use the mean patient weight to calculate a total daily dose. If this is not provided, we will assume that 70 kg is the mean weight. We will not exclude studies on the basis of administered cointerventions.

#### Comparator

At least 1 arm in included trials must not receive parenteral vitamin C; that arm may receive either placebo or active treatment. We will exclude studies that compare the same regimen of parenteral vitamin C with different cointerventions across multiple arms and studies that compare different regimens of parenteral vitamin C without an arm that lacks parenteral vitamin C administration.

#### Primary Outcomes

The primary outcomes will include in-hospital mortality, 30-day mortality, and 90-day mortality. We will include mortality data that are reported closest to the time points of interest.

#### Secondary Outcomes

The secondary outcomes will include the need for and duration of intensive care unit admission and invasive ventilation; the duration of hospitalization; the time to clinical improvement based on changes in the WHO 7-point ordinal scale for clinical status [26] (or another severity measure); changes in SOFA scores from baseline; stage 3 acute kidney injury, as determined by the Kidney Disease: Improving Global Outcomes criteria [27]; and the need for renal replacement therapy. Other secondary outcomes will be serious adverse events that result in the discontinuation of vitamin C, new hemolysis, new nephrolithiasis or clinically important oxaluria, and hypoglycemic episodes. The time point of interest for all secondary outcomes will be the 30-day follow-up or the closest available time point.

We will include studies meeting the eligibility criteria and reporting at least 1 primary or secondary outcome of interest. We will include grey literature and conference abstracts meeting other eligibility criteria.

### Study Selection

Paired reviewers will screen identified citations at the title and abstract screening level based on predefined eligibility criteria. Potentially eligible citations will subsequently be reviewed at the full-text screening level by paired reviewers. Screening will be completed independently and in duplicate. Disagreements will be resolved by a third reviewer.

### Data Extraction and Risk of Bias Assessment

We will extract data on study populations, interventions, comparators, and outcomes of interest as well as conduct prespecified subgroup analyses. Specifically, we will extract data on the following variables:

- Study-level characteristics: publication status, study design, and funding type
- Patient-level baseline demographic characteristics: country, median or mean age, proportion of males, and proportion of patients with renal disease
- Patient-level baseline clinical characteristics: primary infectious source (pneumonia vs intra-abdominal vs genitourinary vs other), proportion of intensive care unit–admitted patients, proportion of patients with confirmed COVID-19, illness severity score, proportion of patients needing renal replacement therapy, proportion of patients needing vasopressors, proportion of patients with ARDS, proportion of patients needing basic supplemental oxygen modalities (including face masks, nasal prongs, noninvasive ventilation, and high-flow nasal cannulas), proportion of patients needing invasive mechanical ventilation, and mean or median serum lactate level
- Study-level intervention arm characteristics: description of regimen, number of patients randomized, total daily vitamin C dose, dosing level (as defined in the *Intervention* section), duration of treatment, cointerventions (including thiamine, corticosteroids, and other therapies) and associated doses and durations, route of administration, mean time from presentation or enrollment to the initiation of the intervention, mean fluid volume administered from admission or sepsis recognition to randomization, and mean fluid volume administered from randomization up to the first 24 hours postrandomization and received in first 6 hours of therapy
- Study-level dichotomous outcomes: outcomes reported, follow-up time (in days), number of participants analyzed, and number of events reported
- Study-level continuous outcomes: outcomes reported, follow-up time (in days), number of participants analyzed, measures and estimates of central tendency, measures and estimates of variability, and definitions used for time to clinical improvement outcomes (if applicable)
- Study-level subgroup analyses (see *Planned Subgroup and Sensitivity Analyses* section)
- Outcome-level risk of bias (RoB) assessment: dichotomous or continuous outcomes (1 evaluation per outcome) and assessment by domain, with justification

We will use a modified version of the RoB 2.0 tool, which was implemented in a recent systematic review [11], for related assessments in every eligible study. The RoB will be classified as “low,” “probably low,” “probably high,” or “high” for the following domains: bias due to randomization, bias due to deviations from the intended intervention, bias due to missing outcome data, bias in the measurement of the outcome, bias in the selection of the reported result, and other biases. We will rate the overall RoB based on the highest risk attributed to any criterion.

Paired reviewers will extract data and evaluate the RoB of eligible studies independently and in duplicate. Disagreements will be resolved by consensus. Data extraction and RoB evaluations will be conducted by using predetermined forms.

### Assessment of the Certainty of the Evidence

We will assess the overall certainty of the evidence for each outcome by using the Grading of Recommendations Assessment, Development and Evaluation (GRADE) framework, based on the following domains: RoB, imprecision, inconsistency, indirectness, and publication bias. The overall certainty of evidence will be rated as “very low,” “low,” “moderate,” or “high.” We will consider rating down the certainty of evidence for the RoB domain based on a lack of blinding for subjective outcomes only. We will make judgments on imprecision by using a minimally contextualized approach. We will consider any CI encompassing the null effect to be imprecise while taking into consideration important and trivial effects [28].

### Statistical Analyses

We plan to conduct random effects modeling for our meta-analyses. All analyses will be performed in RevMan 5.3 (Cochrane Collaboration). Study weights will be generated by using the inverse variance method. We will present dichotomous outcomes as RRs and continuous outcomes as mean differences or standardized mean differences; these will all be presented with 95% CIs. We will assume a normal distribution for continuous outcomes and will convert interquartile ranges to standard deviations, as per the guidance from the Cochrane Collaboration [29]. We will use a web-based plot digitizer to obtain estimates only when outcomes of interest are presented graphically.

We will assess statistical heterogeneity among studies by using the *I*² measure, and inconsistency will be judged with GRADE assessments on the basis of the magnitude and direction of heterogeneity. We will assess publication bias via the visual inspection of funnel plots. This method will be supplemented by a regression test if at least 10 studies contribute to an outcome [30].

For each outcome, the primary analysis will include data from all studies meeting the eligibility criteria and reporting the outcome.

### Planned Subgroup and Sensitivity Analyses

If sufficient data are available, we will plan the following subgroup analyses: (1) high-dose vitamin C versus moderate-dose vitamin C versus low-dose vitamin C (as previously defined; hypothesis: greater effects in the high-dose group), (2) vitamin C administration as monotherapy versus vitamin C administration in combination with other interventions (hypothesis: no differences in effects), and (3) treatment of patients with COVID-19 versus treatment of other severe infections (hypothesis: no differences in effects).

We will plan sensitivity analyses, which will be limited to (1) peer-reviewed studies published in full text and (2) low RoB studies.

### Patient and Public Involvement

We do not plan to involve patients or public members in the conduct of this systematic review.

### Dissemination

We plan to disseminate our study results through national and international critical care–focused and methodology-focused conferences. We also plan to publish our findings in a peer-reviewed journal.

### Ongoing Updates

The review will be a living meta-analysis and will be iteratively updated every 4 to 6 months to incorporate emerging randomized trial evidence. Meta-analyses will be updated iteratively with the same frequency. When new evidence changes the certainty or direction of estimates for any primary or secondary outcomes, we will disseminate subsequent iterations of our findings.

## Results

Among the 1386 citations identified as of March 30, 2021, a total of 17 eligible RCTs have been identified as of September 2021. We are in the process of updating the search strategy and associated data analyses.

## Discussion

Severe infections that result in hospital admission and organ dysfunction in sepsis are associated with significant global morbidity and mortality [3]. The COVID-19 pandemic has resulted in over 251 million COVID-19 cases and 5 million deaths [31]. Given the publication of recent trials and a variety of tested vitamin C regimens, an updated evidence synthesis regarding the efficacy and safety of parenteral vitamin C in this context is warranted. Our findings will inform clinical practices in hospitals for the management of severe infections, including COVID-19.

Our planned systematic review has a number of strengths. First, we will summarize evidence relevant to all severe infections for which hospitalization is required. Our findings will therefore be more applicable than those of recent reviews on vitamin C in critically ill patients in general [17]. Second, we will incorporate a number of recent landmark trials that have been published in the interim, including the CITRIS-ALI (Vitamin C Infusion for Treatment in Sepsis-Induced Acute Lung Injury) trial [18], VITAMINS (Vitamin C, Hydrocortisone and Thiamine in Patients With Septic Shock) trial [19], ATESS (Ascorbic Acid and Thiamine Effect in Septic Shock) trial [20], ORANGES (Outcomes of Metabolic Resuscitation Using Ascorbic Acid, Thiamine, and Glucocorticoids in the Early Treatment of Sepsis) trial [32], HYVCTTSSS (Hydrocortisone, Vitamin C, and Thiamine for the Treatment of Sepsis and Septic Shock) trial [33], and ACTS (Ascorbic Acid, Corticosteroids, and Thiamine in Sepsis and Septic Shock) trial [34]. These trials will provide higher certainty estimates for our primary and secondary outcomes of interest. Third, we intend to create regular updates to produce a living systematic review that will provide updated estimates of effects as new trial evidence becomes available. Fourth, we have used a comprehensive search strategy that was developed with the aid of a medical librarian; have incorporated both published and unpublished data; have a predefined review, statistical analysis, and subgroup analysis plan in place; and intend to use the GRADE methodology to systematically evaluate certainty in effect estimates. Fifth, our summary of ongoing trials, which will be kept updated on an iterative basis, will provide a useful resource that contains upcoming and emerging evidence. Finally, we intend to share our data with any interested living network meta-analysis teams, so that evidence on parenteral vitamin C administration may be used to iteratively inform indirect comparisons to other treatments for severe infection, sepsis, and COVID-19.

In conclusion, this protocol describes the detailed methodology of a planned living systematic review and meta-analysis that will address the comparative efficacy and safety of parenteral vitamin C administration in patients with severe infections. The results will be of importance to critical care physicians and hospitalists who manage severe infection in daily practice, and they may directly inform international clinical guidance regarding the management of sepsis.

## Appendix

Multimedia Appendix 1 Preferred Reporting Items for Systematic Reviews and Meta-Analysis Protocols (PRISMA-P) checklist.

Multimedia Appendix 2 Search strategy.

## Abbreviations

ACTS: Ascorbic Acid, Corticosteroids, and Thiamine in Sepsis and Septic Shock
ARDS: acute respiratory distress syndrome
ATESS: Ascorbic Acid and Thiamine Effect in Septic Shock
CI: Confidence Interval
CITRIS-ALI: Vitamin C Infusion for Treatment in Sepsis-Induced Acute Lung Injury
GRADE: Grading of Recommendations Assessment, Development and Evaluation
HYVCTTSSS: Hydrocortisone, Vitamin C, and Thiamine for the Treatment of Sepsis and Septic Shock
ORANGES: Outcomes of Metabolic Resuscitation Using Ascorbic Acid, Thiamine, and Glucocorticoids in the Early Treatment of Sepsis
PRISMA-P: Preferred Reporting Items for Systematic Reviews and Meta-Analysis Protocols
RCT: randomized controlled trial
RoB: risk of bias
RR: risk ratio
SOFA: sepsis-related organ failure assessment
VITAMINS: Vitamin C, Hydrocortisone and Thiamine in Patients With Septic Shock
WHO: World Health Organization

## References

[1] Vincent JL, Moreno R, Takala J, Willatts S, De Mendonça A, Bruining H, Reinhart CK, Suter PM, Thijs LG (1996). The SOFA (Sepsis-related Organ Failure Assessment) score to describe organ dysfunction/failure. On behalf of the Working Group on Sepsis-Related Problems of the European Society of Intensive Care Medicine. Intensive Care Med.

[2] Singer M, Deutschman CS, Seymour CW, Shankar-Hari M, Annane D, Bauer M, Bellomo R, Bernard GR, Chiche JD, Coopersmith CM, Hotchkiss RS, Levy MM, Marshall JC, Martin GS, Opal SM, Rubenfeld GD, van der Poll T, Vincent J, Angus DC (2016). The Third International Consensus Definitions for Sepsis and Septic Shock (Sepsis-3). JAMA.

[3] Rudd KE, Johnson SC, Agesa KM, Shackelford KA, Tsoi D, Kievlan DR, Colombara DV, Ikuta KS, Kissoon N, Finfer S, Fleischmann-Struzek C, Machado FR, Reinhart KK, Rowan K, Seymour CW, Watson RS, West TE, Marinho F, Hay SI, Lozano R, Lopez AD, Angus DC, Murray CJL, Naghavi M (2020). Global, regional, and national sepsis incidence and mortality, 1990-2017: analysis for the Global Burden of Disease Study. Lancet.

[4] Rhodes A, Evans LE, Alhazzani W, Levy MM, Antonelli M, Ferrer R, Kumar A, Sevransky JE, Sprung CL, Nunnally ME, Rochwerg B, Rubenfeld GD, Angus DC, Annane D, Beale RJ, Bellinghan GJ, Bernard GR, Chiche J, Coopersmith C, De Backer DP, French CJ, Fujishima S, Gerlach H, Hidalgo JL, Hollenberg SM, Jones AE, Karnad DR, Kleinpell RM, Koh Y, Lisboa TC, Machado FR, Marini JJ, Marshall JC, Mazuski JE, McIntyre LA, McLean AS, Mehta S, Moreno RP, Myburgh J, Navalesi P, Nishida O, Osborn TM, Perner A, Plunkett CM, Ranieri M, Schorr CA, Seckel MA, Seymour CW, Shieh L, Shukri KA, Simpson SQ, Singer M, Thompson BT, Townsend SR, Van der Poll T, Vincent JL, Wiersinga WJ, Zimmerman JL, Dellinger RP (2017). Surviving sepsis campaign: International guidelines for management of sepsis and septic shock: 2016. Crit Care Med.

[5] Seymour CW, Gesten F, Prescott HC, Friedrich ME, Iwashyna TJ, Phillips GS, Lemeshow S, Osborn T, Terry KM, Levy MM (2017). Time to treatment and mortality during mandated emergency care for sepsis. N Engl J Med.

[6] Rochwerg B, Oczkowski SJ, Siemieniuk RAC, Agoritsas T, Belley-Cote E, D'Aragon F, Duan E, English S, Gossack-Keenan K, Alghuroba M, Szczeklik W, Menon K, Alhazzani W, Sevransky J, Vandvik PO, Annane D, Guyatt G (2018). Corticosteroids in sepsis: An updated systematic review and meta-analysis. Crit Care Med.

[7] Annane D, Bellissant E, Bollaert PE, Briegel J, Keh D, Kupfer Y, Pirracchio R, Rochwerg B (2019). Corticosteroids for treating sepsis in children and adults. Cochrane Database Syst Rev.

[8] Lamontagne F, Rochwerg B, Lytvyn L, Guyatt GH, Møller MH, Annane D, Kho ME, Adhikari NKJ, Machado F, Vandvik PO, Dodek P, Leboeuf R, Briel M, Hashmi M, Camsooksai J, Shankar-Hari M, Baraki MK, Fugate K, Chua S, Marti C, Cohen D, Botton E, Agoritsas T, Siemieniuk RAC (2018). Corticosteroid therapy for sepsis: a clinical practice guideline. BMJ.

[9] Ye Z, Rochwerg B, Wang Y, Adhikari NK, Murthy S, Lamontagne F, Fowler RA, Qiu H, Wei L, Sang L, Loeb M, Shen N, Huang M, Jiang Z, Arabi YM, Colunga-Lozano LE, Jiang L, Koh Y, Liu D, Liu F, Phua J, Shen A, Huo T, Du B, Zhai S, Guyatt GH (2020). Treatment of patients with nonsevere and severe coronavirus disease 2019: an evidence-based guideline. CMAJ.

[10] Lamontagne F, Agoritsas T, Siemieniuk R, Rochwerg B, Bartoszko J, Askie L, Macdonald H, Amin W, Bausch FJ, Burhan E, Cecconi M, Chanda D, Dat VQ, Du B, Geduld H, Gee P, Nerina H, Hashimi M, Hunt BJ, Kabra S, Kanda S, Kawano-Dourado L, Kim YJ, Kissoon N, Kwizera A, Leo YS, Mahaka I, Manai H, Mino G, Nsutebu E, Pshenichnaya N, Qadir N, Ranganathan SS, Sabzwari S, Sarin R, Sharland M, Shen Y, Souza JP, Stegemann M, Ugarte S, Venkatapuram S, Vuyiseka D, Preller J, Brignardello-Petersen R, Kum E, Qasim A, Zeraatkar D, Owen A, Guyatt G, Lytvyn L, Diaz J, Vandvik PO, Jacobs M (2021). A living WHO guideline on drugs to prevent covid-19. BMJ.

[11] Siemieniuk RA, Bartoszko JJ, Ge L, Zeraatkar D, Izcovich A, Kum E, Pardo-Hernandez H, Qasim A, Martinez JPD, Rochwerg B, Lamontagne F, Han MA, Liu Q, Agarwal A, Agoritsas T, Chu DK, Couban R, Cusano E, Darzi A, Devji T, Fang B, Fang C, Flottorp SA, Foroutan F, Ghadimi M, Heels-Ansdell D, Honarmand K, Hou L, Hou X, Ibrahim Q, Khamis A, Lam B, Loeb M, Marcucci M, McLeod SL, Motaghi S, Murthy S, Mustafa RA, Neary JD, Rada G, Riaz IB, Sadeghirad B, Sekercioglu N, Sheng L, Sreekanta A, Switzer C, Tendal B, Thabane L, Tomlinson G, Turner T, Vandvik PO, Vernooij RW, Viteri-García A, Wang Y, Yao L, Ye Z, Guyatt GH, Brignardello-Petersen R (2020). Drug treatments for covid-19: living systematic review and network meta-analysis. BMJ.

[12] Moskowitz A, Andersen LW, Huang DT, Berg KM, Grossestreuer AV, Marik PE, Sherwin RL, Hou PC, Becker LB, Cocchi MN, Doshi P, Gong J, Sen A, Donnino MW (2018). Ascorbic acid, corticosteroids, and thiamine in sepsis: a review of the biologic rationale and the present state of clinical evaluation. Crit Care.

[13] Marik PE, Hooper MH (2018). Doctor-your septic patients have scurvy!. Crit Care.

[14] Oudemans-van Straaten HM, Spoelstra-de Man AM, de Waard MC (2014). Vitamin C revisited. Crit Care.

[15] Chiscano-Camón L, Ruiz-Rodriguez JC, Ruiz-Sanmartin A, Roca O, Ferrer R (2020). Vitamin C levels in patients with SARS-CoV-2-associated acute respiratory distress syndrome. Crit Care.

[16] (2020). A coordinated global research roadmap: 2019 novel coronavirus. World Health Organization.

[17] Langlois PL, Manzanares W, Adhikari NKJ, Lamontagne F, Stoppe C, Hill A, Heyland DK (2019). Vitamin C administration to the critically ill: A systematic review and meta-analysis. JPEN J Parenter Enteral Nutr.

[18] Fowler 3rd AA, Truwit JD, Hite RD, Morris PE, DeWilde C, Priday A, Fisher B, Thacker 2nd LR, Natarajan R, Brophy DF, Sculthorpe R, Nanchal R, Syed A, Sturgill J, Martin GS, Sevransky J, Kashiouris M, Hamman S, Egan KF, Hastings A, Spencer W, Tench S, Mehkri O, Bindas J, Duggal A, Graf J, Zellner S, Yanny L, McPolin C, Hollrith T, Kramer D, Ojielo C, Damm T, Cassity E, Wieliczko A, Halquist M (2019). Effect of vitamin C infusion on organ failure and biomarkers of onflammation and vascular injury in patients with sepsis and severe acute respiratory failure: The CITRIS-ALI randomized clinical trial. JAMA.

[19] Fujii T, Luethi N, Young PJ, Frei DR, Eastwood GM, French CJ, Deane AM, Shehabi Y, Hajjar LA, Oliveira G, Udy AA, Orford N, Edney SJ, Hunt AL, Judd HL, Bitker L, Cioccari L, Naorungroj T, Yanase F, Bates S, McGain F, Hudson EP, Al-Bassam W, Dwivedi DB, Peppin C, McCracken P, Orosz J, Bailey M, Bellomo R, VITAMINS Trial Investigators (2020). Effect of vitamin C, hydrocortisone, and thiamine vs hydrocortisone alone on time alive and free of vasopressor support among patients with septic shock: The VITAMINS randomized clinical trial. JAMA.

[20] Hwang SY, Ryoo SM, Park JE, Jo YH, Jang DH, Suh GJ, Kim T, Kim YJ, Kim S, Cho H, Jo IJ, Chung SP, Choi SH, Shin TG, Kim WY, Korean Shock Society (KoSS) (2020). Combination therapy of vitamin C and thiamine for septic shock: a multi-centre, double-blinded randomized, controlled study. Intensive Care Med.

[21] Murthy S, Leligdowicz A, Adhikari NKJ (2015). Intensive care unit capacity in low-income countries: a systematic review. PLoS One.

[22] Seymour CW, Liu VX, Iwashyna TJ, Brunkhorst FM, Rea TD, Scherag A, Rubenfeld G, Kahn JM, Shankar-Hari M, Singer M, Deutschman CS, Escobar GJ, Angus DC (2016). Assessment of clinical criteria for sepsis: For the Third International Consensus Definitions for Sepsis and Septic Shock (Sepsis-3). JAMA.

[23] World Health Organization (2011). Hospital Care for Adolescents and Adults: Guidelines for the Management of Common Illnesses With Limited Resources.

[24] Fernando SM, Tran A, Taljaard M, Cheng W, Rochwerg B, Seely AJE, Perry JJ (2018). Prognostic accuracy of the quick sequential organ failure assessment for mortality in patients with suspected infection: A systematic review and meta-analysis. Ann Intern Med.

[25] Yanase F, Fujii T, Naorungroj T, Belletti A, Luethi N, Carr AC, Young PJ, Bellomo R (2020). Harm of IV high-dose vitamin C therapy in adult patients: A scoping review. Crit Care Med.

[26] R&D blueprint and COVID-19. World Health Organization.

[27] (2012). KDIGO Clinical Practice Guideline for Acute Kidney Injury. Kidney Disease: Improving Global Outcomes.

[28] Hultcrantz M, Rind D, Akl EA, Treweek S, Mustafa RA, Iorio A, Alper BS, Meerpohl JJ, Murad MH, Ansari MT, Katikireddi SV, Östlund P, Tranæus S, Christensen R, Gartlehner G, Brozek J, Izcovich A, Schünemann H, Guyatt G (2017). The GRADE Working Group clarifies the construct of certainty of evidence. J Clin Epidemiol.

[29] Wan X, Wang W, Liu J, Tong T (2014). Estimating the sample mean and standard deviation from the sample size, median, range and/or interquartile range. BMC Med Res Methodol.

[30] Sterne JAC, Sutton AJ, Ioannidis JPA, Terrin N, Jones DR, Lau J, Carpenter J, Rücker G, Harbord RM, Schmid CH, Tetzlaff J, Deeks JJ, Peters J, Macaskill P, Schwarzer G, Duval S, Altman DG, Moher D, Higgins JPT (2011). Recommendations for examining and interpreting funnel plot asymmetry in meta-analyses of randomised controlled trials. BMJ.

[31] Coronavirus resource center. Johns Hopkins Univeristy & Medicine.

[32] Iglesias J, Vassallo AV, Patel VV, Sullivan JB, Cavanaugh J, Elbaga Y (2020). Outcomes of metabolic resuscitation using ascorbic acid, thiamine, and glucocorticoids in the early treatment of sepsis: The ORANGES trial. Chest.

[33] Chang P, Liao Y, Guan J, Guo Y, Zhao M, Hu J, Zhou J, Wang H, Cen Z, Tang Y, Liu Z (2020). Combined treatment with hydrocortisone, vitamin C, and thiamine for sepsis and septic shock: A randomized controlled trial. Chest.

[34] Moskowitz A, Huang DT, Hou PC, Gong J, Doshi PB, Grossestreuer AV, Andersen LW, Ngo L, Sherwin RL, Berg KM, Chase M, Cocchi MN, McCannon JB, Hershey M, Hilewitz A, Korotun M, Becker LB, Otero RM, Uduman J, Sen A, Donnino MW, ACTS Clinical Trial Investigators (2020). Effect of ascorbic acid, corticosteroids, and thiamine on organ injury in septic shock: The ACTS randomized clinical trial. JAMA.

