# Supplementary material for: Administration of Parenteral Vitamin C in Patients With Severe Infection: Protocol for a Systematic Review and Meta-analysis
Source: JMIR Res Protoc. 2022 Jan 6;11(1):e33989. doi: 10.2196/33989 (PMC8734609; doi:10.2196/33989)
Supplement: Multimedia Appendix 2 [file resprot_v11i1e33989_app2.pdf]

Multimedia Appendix 2: Search strategy

Number of hits reported are from initial search conducted in September 2020 (prior to search updates).

1. OVID MEDLINE

Database: OVID Medline Epub Ahead of Print, In-Process & Other Non-Indexed Citations, Ovid MEDLINE(R) Daily and Ovid MEDLINE(R) 1946 to Present
Search Strategy:
----
1 exp Ascorbic Acid/ (42344)
2 exp Vitamins/ and C.mp. (44095)
3 (ascorb* or vitc).mp. [mp=title, abstract, original title, name of substance word, subject heading word, floating sub-heading word, keyword heading word, organism supplementary concept word, protocol supplementary concept word, rare disease supplementary concept word, unique identifier, synonyms] (67045)
4 vitamin C.mp. [mp=title, abstract, original title, name of substance word, subject heading word, floating sub-heading word, keyword heading word, organism supplementary concept word, protocol supplementary concept word, rare disease supplementary concept word, unique identifier, synonyms] (24009)
5 (vit* adj C).mp. (25951)
6 or/1-5 (101360)
7 exp sepsis/ or shock, septic/ (123787)
8 (sepsis or septic).mp. [mp=title, abstract, original title, name of substance word, subject heading word, floating sub-heading word, keyword heading word, organism supplementary concept word, protocol supplementary concept word, rare disease supplementary concept word, unique identifier, synonyms] (167963)
9 Multiple Organ Failure/ (10938)
10 Critical Illness/ (29338)
11 Respiratory Distress Syndrome, Adult/ (19714)
12 ((severe or serious or critical or intensive) adj3 (illness or infection* or shock or care)).mp. [mp=title, abstract, original title, name of substance word, subject heading word, floating sub-heading word, keyword heading word, organism supplementary concept word, protocol supplementary concept word, rare disease supplementary concept word, unique identifier, synonyms] (294745)
13 ICU.mp. (57952)
14 (organ adj3 failure).mp. [mp=title, abstract, original title, name of substance word, subject heading word, floating sub-heading word, keyword heading word, organism supplementary concept word, protocol supplementary concept word, rare disease supplementary concept word, unique identifier, synonyms] (27855)
15 or/7-12 (488021)
16 6 and 15 (821)
17 exp Parenteral Nutrition/ (24006)
18 exp Administration, Intravenous/ (143297)
19 (intravenous or parenteral* or enteral* or IV or infus* or transfus* or inject*).mp. (1869924)
20 or/17-19 (1869924)
21 16 and 20 (276)

2. EMBASE

Database: Embase <1974 to 2020 September 10>
Search Strategy:
----
1 exp ascorbic acid/ (91239)
2 exp vitamin/ (630902)
3 exp vitamin/ and C.mp. (90708)
4 (vit* adj C).mp. [mp=title, abstract, heading word, drug trade name, original title, device manufacturer, drug manufacturer, device trade name, keyword, floating subheading word, candidate term word] (29114)
5 or/1-4 (634724)
6 exp sepsis/ or exp septic shock/ (266658)
7 (sepsis or septic).mp. [mp=title, abstract, heading word, drug trade name, original title, device manufacturer, drug manufacturer, device trade name, keyword, floating subheading word, candidate term word] (272661)
8 multiple organ failure/ (38757)
9 exp critical illness/ (29817)
10 adult respiratory distress syndrome/ (38485)
11 ((severe or serious or critical or intensive) adj3 (illness or infection* or shock or care)).mp. [mp=title, abstract, heading word, drug trade name, original title, device manufacturer, drug manufacturer, device trade name, keyword, floating subheading word, candidate term word] (475971)
12 ICU.mp. (119275)
13 (organ adj3 failure).mp. [mp=title, abstract, heading word, drug trade name, original title, device manufacturer, drug manufacturer, device trade name, keyword, floating subheading word, candidate term word] (64554)
14 or/6-13 (813307)
15 5 and 14 (12343)
16 exp parenteral nutrition/ (49030)
17 exp fluid therapy/ (93508)
18 exp intravenous drug administration/ (357987)
19 (intravenous or parenteral* or enteral* or IV or infus* or transfus* or inject*).mp. [mp=title, abstract, heading word, drug trade name, original title, device manufacturer, drug manufacturer, device trade name, keyword, floating subheading word, candidate term word] (2952821)
20 or/16-19 (2978687)
21 15 and 20 (6239)
22 randomized controlled trial/ (619164)
23 Controlled clinical study/ (464566)
24 random$.ti,ab. (1570722)
25 randomization/ (87825)
26 intermethod comparison/ (263313)
27 placebo.ti,ab. (310598)
28 (compare or compared or comparison).ti. (516864)
29 ((evaluated or evaluate or evaluating or assessed or assess) and (compare or compared or comparing or comparison)).ab. (2161229)
30 (open adj label).ti,ab. (81079)
31 ((double or single or doubly or singly) adj (blind or blinded or blindly)).ti,ab. (235337)
32 double blind procedure/ (175711)
33 parallel group$1.ti,ab. (26025)

34 (crossover or cross over).ti,ab. (106595)
35 ((assign$ or match or matched or allocation) adj5 (alternate or group$1 or intervention$1 or patient$1 or subject$1 or participant$1)).ti,ab. (336261)
36 (assigned or allocated).ti,ab. (395805)
37 (controlled adj7 (study or design or trial)).ti,ab. (356466)
38 (volunteer or volunteers).ti,ab. (249370)
39 human experiment/ (511875)
40 trial.ti. (307823)
41 or/22-40 (5111625)
42 (random$ adj sampl$ adj7 ("cross section$" or questionnaire$1 or survey$ or database$1)).ti,ab. not (comparative study/ or controlled study/ or randomi?ed controlled.ti,ab. or randomly assigned.ti,ab.) (8176)
43 Cross-sectional study/ not (randomized controlled trial/ or controlled clinical study/ or controlled study/ or randomi?ed controlled.ti,ab. or control group$1.ti,ab.) (243957)
44 (((case adj control$) and random$) not randomi?ed controlled).ti,ab. (17560)
45 (Systematic review not (trial or study)).ti. (151851)
46 (nonrandom$ not random$).ti,ab. (16349)
47 "Random field$".ti,ab. (2352)
48 (random cluster adj3 sampl$).ti,ab. (1302)
49 (review.ab. and review.pt.) not trial.ti. (823551)
50 "we searched".ab. and (review.ti. or review.pt.) (33158)
51 "update review".ab. (109)
52 (databases adj4 searched).ab. (37504)
53 (rat or rats or mouse or mice or swine or porcine or murine or sheep or lambs or pigs or piglets or rabbit or rabbits or cat or cats or dog or dogs or cattle or bovine or monkey or monkeys or trout or marmoset$1).ti. and animal experiment/ (1076744)
54 Animal experiment/ not (human experiment/ or human/) (2268329)
55 or/42-54 (3529703)
56 41 not 55 (4547434)
57 21 and 56 (1021)

3. Cochrane Central Register of Controlled Trials

Search Name: Vitamin C
Comment:

ID	Search	Hits
#1	MeSH descriptor: [Ascorbic Acid] explode all trees	2210
#2	MeSH descriptor: [Vitamins] explode all trees	4661
#3	C	358838
#4	#2 and #3	1322
#5	vitamin C	10679
#6	vit* NEAR C	5743
#7	#1 or #4 or #4 or #6	7258
#8	MeSH descriptor: [Sepsis] explode all trees	4496
#9	sepsis or septic	14115
#10	MeSH descriptor: [Multiple Organ Failure] explode all trees	408
#11	MeSH descriptor: [Critical Illness] explode all trees	2233
#12	MeSH descriptor: [Respiratory Distress Syndrome, Adult] explode all trees	1347
#13	((severe or serious or critical or intensive) NEAR/3 (illness or infection* or shock or care))	54323
#14	ICU	12766
#15	organ NEAR/3 failure	3608
#16	#8 or #9 or #10 or #11 or #12 or #13 or #14 or #15	69648
#17	#7 and #16	454
#18	MeSH descriptor: [Parenteral Nutrition] explode all trees	1649
#19	MeSH descriptor: [Administration, Intravenous] explode all trees	18479
#20	intravenous or parenteral* or enteral* or IV or infus* or transfus* or inject*	266767
#21	#18 or #19 or #20	266767
#22	#17 AND #21 in Trials	175

4. CINAHL

#	Query	Results
S22	S17 AND S21	148
S21	S18 OR S19 OR S20	295,942
S20	TX (intravenous or parenteral* or enteral* or IV or infus* or transfus* or inject*)	295,930
S19	(MH "Administration, Intravenous+")	8,584
S18	(MH "Parenteral Nutrition+")	7,037
S17	S7 AND S16	434
S16	S8 OR S9 OR S10 OR S11 OR S12 OR S13 OR S14 OR S15	313,921
S15	TX organ N3 failure	6,062
S14	"ICU"	36,340
S13	TX ((severe or serious or critical or intensive) N3 (illness or infection* or shock or care))	282,616
S12	(MH "Respiratory Distress Syndrome, Acute")	7,701
S11	(MH "Critical Illness")	12,439
S10	(MH "Multiple Organ Dysfunction Syndrome+")	15,989
S9	TX sepsis or septic	38,025
S8	(MH "Sepsis") OR (MH "Shock, Septic")	20,556
S7	S1 OR S4 OR S5 OR S6	9,494
S6	"vitamin C"	5,163
S5	TX ascorb* or vitc	6,750
S4	S2 AND S3	1,361
S3	TX C	799,780
S2	(MH "Vitamins")	8,189
S1	(MH "Ascorbic Acid")	5,648

5. Centers for Disease Control COVID-19 Database

Search conducted from:
https://www.cdc.gov/library/researchguides/2019novelcoronavirus/researcharticles.html

Imported: 107514 articles.

Keyword search: vitamin OR ascorb*

Filtered for RCTs using RobotReviewer (https://robotsearch.vortext.systems/): 123 articles.
